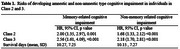# Associations between instrumental activities of daily living and the risk for memory and non‐memory related cognitive impairment in older adults

**DOI:** 10.1002/alz70860_106837

**Published:** 2025-12-23

**Authors:** Oshadi M Jayakody, Helena M Blumen, Cuiling Wang, Ying Jin

**Affiliations:** ^1^ Albert Einstein College of Medicine, Bronx, NY, USA; ^2^ Stony Brook Medicine, Stony Brook, NY, USA; ^3^ Department of Epidemiology and Population Health, Department of Neurology, Albert Einstein School of Medicine, New York, USA, Bronx, NY, USA

## Abstract

**Background:**

Alzheimer's disease and related dementias (ADRD) develop over 20 years with subtle impairments in Instrumental Activities of Daily Living (IADLs). This study aimed to determine whether specific IADLs are associated with risks for cognitive impairment in pre‐ADRD stages.

**Methods:**

We used Gateway to Global Aging Data (g2aging.org) from the US Health and Retirement Study. In adults ≥65 years without an ADRD diagnosis (*n* = 10, 346), IADL impairments were self‐reported as difficulty in managing money, medication, reading maps, using telephone, shopping for groceries and preparing meals. Memory‐related cognitive impairment was defined as ≥1.5 SD below the study‐specific mean for total recall combined with poor self‐reported memory. Non‐memory‐related cognitive impairment was ≥1.5 SD below the mean in ≥2 cognitive tests including orientation, serial 7s and backward counting. Latent class analysis was performed to identify different groups of IADLs at baseline. Cox proportional hazards models adjusted for age, sex, education, were used to determine the associations between group membership in IADLs and the risk for cognitive impairment. Models were adjusted for post‐stratification sample weights.

**Results:**

Weighted mean age of participants was 74.3 years (SD 6.8), and 58% were women. Three IADL groups were identified: A high functioning group (86%), with low probabilities of difficulty in any IADLs ; a group with moderate difficulty in specific tasks such as managing money, shopping, reading maps and making meals (10%) ; and a low functioning group (4%), with high difficulty across all IADLs. The low functioning group showed increased risk (hazard ratio [HR] 2‐2.5; *p* <0.001) of developing both types of cognitive impairment compared to the high functioning group (during a mean 10.3 (SD 7.2) years of follow‐up). The moderate functioning group also showed an increased risk for memory‐ (HR 2.0, *p* = 0.001) and non‐memory‐related impairment (HR 1.6, *p* <0.001) compared to the high functioning group (Table 1).

**Conclusions:**

Our findings suggest that even moderate difficulty in performing specific IADLs (i.e., managing money) is associated with an increased risk for cognitive impairment in older adults. These IADLs may serve as early risk indicators and have the potential to be developed into clinical trial endpoints in pre‐clinical ADRD populations.